# Use of thyroid hormones in hypothyroid and euthyroid patients: a 2022 THESIS questionnaire survey of members of the Latin American Thyroid Society (LATS)

**DOI:** 10.1186/s13044-023-00182-4

**Published:** 2023-09-29

**Authors:** Jessica F. Cassemiro, Veronica Ilera, Stella Batalles, Adriana Reyes, Endre V. Nagy, Enrico Papini, Petros Perros, Laszlo Hegedüs, Helton Estrela Ramos

**Affiliations:** 1 Department of Bioregulation, Health & Science Institute, Federal University of Bahia, Avenida Reitor Miguel Calmon, S/N. Vale do Canela. Room 325, Salvador, Bahia Brazil; 2 Department of Endocrinology, Hospital Ramos Mejía, Caba, Argentina; 3 Cardiovascular Institute of Rosario, Santa Fe, Argentina; 4 Division of Endocrinology, Department of Medicine, Faculty of Medicine, University of Debrecen, Debrecen, Hungary; 5 Department of Endocrinology and Metabolism, Regina Apostolorum Hospital, Rome, Italy; 6 Department of Endocrinology, Royal Victoria Infirmary, Newcastle upon Tyne, UK; 7 Department of Endocrinology, Odense University Hospital, Odense, Denmark

**Keywords:** Levothyroxine, Liothyronine, Hypothyroidism, Euthyroidism, Survey, Latin American Thyroid Society

## Abstract

**Purpose:**

Inconsistencies in the medical management of hypothyroidism have been reported between endocrinologists in different countries. This study aimed to identify the attitudes of Latin America thyroid specialists towards the use of thyroid hormones.

**Methods:**

Online survey of members of the Latin America Thyroid Society.

**Results:**

81/446 (18.2%) completed the questionnaire. Levothyroxine (LT4) was the initial treatment of choice for all respondents. 56.8% would consider LT4 use in biochemically euthyroid patients: infertile women with elevated anti-thyroid antibodies (46.9%), resistant depression (17.3%) and growing goiter (12%). Most respondents preferred tablets (39.5%) over liquid formulations (21.0%) or soft gel capsules (22.2%) and would not consider switching formulations in patients with persistent symptoms. 39.5% would never use LT4 + liothyronine (LT3) combination therapy in symptomatic euthyroid patients, due to low quality evidence for benefit. 60.5% reported that persistence of symptoms despite normal TSH is rare (below 5% of patients) and its prevalence has been stable over the last five years. Psychosocial factors (84.0%), comorbidities (86.4%) and the patient unrealistic expectation (72.8%) were considered the top three explanations for this phenomenon.

**Conclusion:**

LT4 tablets is the treatment of choice for hypothyroidism. A significant proportion of respondents would use LT4 in some groups of euthyroid individuals, contrasting the recommendations of the major clinical practice guideline indications. LT4 + LT3 combination treatment in euthyroid symptomatic patients was considered by nearly 50%. Practices based on weak or absent evidence included use of thyroid hormones for euthyroid subjects by 56.8% of respondents and use of LT4 + LT3 treatment by 60.5% of respondents for patients with persistent symptoms. In contrast to many European countries, LATS respondents report a low and unchanged proportion of dissatisfied patients over the last five years.

## Introduction

Hypothyroidism is a common condition and together with diabetes constitute the most frequent endocrinopathies. Due to considerable variation in presentation and lack of symptom specificity, the diagnosis of hypothyroidism is biochemical. Measurement of thyroid-stimulating hormone (TSH) along with free thyroxine (FT4) concentrations establish the diagnosis [1]. However, controversy regarding the appropriate TSH cut-off values remains [2].

The prevalence of hypothyroidism is approximately 5% [3]. In Latin America, the Brazilian Longitudinal Study of Adult Health (ELSA-Brazil) examined 9,705 participants with normal thyroid function at baseline that were followed-up over a 4-year period. The incidence of all overt and subclinical thyroid disease was 6.7% (1.73%/year): 1.98% for overt hypothyroidism (0.51%/year), and 3.99% for subclinical hypothyroidism (1.03%/year) [4]. Another study, now analyzing treatment of hypothyroidism, showed that a significant number of patients taking thyroid hormones in Brazil were not in the therapeutic range (28.3% were undertreated and 14.4% were overtreated) [5].

The 2013 Latin American Thyroid Society (LATS) Clinical Practice Guidelines for the management of hypothyroidism recommends LT4 as the only treatment for hypothyroidism [6]. Combination therapy of LT4 and liothyronine (LT3) may be considered in various clinical scenarios such as persistent symptoms despite adequate thyroid hormone replacement with LT4. However, this approach raises concerns of possible iatrogenic thyrotoxicosis and the resulting increase in morbidity and mortality [5–10]. The recently published statement from the Thyroid Department of the Brazilian Society of Endocrinology and Metabolism (SBEM) recommended avoidance of combined therapy with LT4 + LT3 [11]. This recommendation aligns well with the lack of demonstrating significant durable quality of life improvements with LT4 and LT3 combination therapy, or from therapy with desiccated thyroid hormone from a multitude of randomized controlled trials and meta-analyses [12]. It must also be said that those trials also presented some limitations, such as small sample size, low sensitivity of some of the neurocognitive tests and biochemical measures, and therefore results may be inconclusive [13].

Numerous conditions, such as ageing and pregnancy, cause alterations in LT4 requirements [1], as do gastrointestinal diseases, interaction with supplements or medications that interfere with LT4 absorption [2, 14]. Recently, various LT4 formulations (soft-gel capsules or liquid solution) have become available for substitution treatment, to improve bioavailability by facilitating absorption. However, these new formulations are more expensive and are not commercially available in several countries [15].

LATS was invited to participate in an international initiative referred to as THESIS (Treatment of Hypothyroidism in Europe by Specialists an International Survey), which assessed the attitude of experts towards the use of thyroid hormones in hypothyroidism and several euthyroid conditions in 28 countries. So far, 20 national THESIS investigations have been published [16–35].

## Methods

We used a web-based survey constructed with *Google Forms*®, a free-access platform. The questionnaire included 32 questions. Eight were related to the main characteristics of the respondents and 24 to the use of thyroid hormone. Survey completion took 5–10 min. The questionnaire was translated from English into Portuguese and Spanish. The Portuguese version was sent to the Brazilian members and the Spanish one, to the remaining members of LATS. Only respondents that completed the whole questionnaire were able to send it for analysis (that is a configuration of Google forms’ platform).

Between November 18th 2021 and April 18th 2022, an e-mail invitation and five reminders, with an electronic link to the survey, were sent to all persons registered to receive the LATS newsletter. The anonymized responses were stored electronically. Repeat submission from the same IP address was blocked. This project was approved by the Ethical Committee of the Institute of Health Sciences of the Federal University of Bahia (ICS - UFBA), report 5,106,900; CAAE number 52980621.5.0000.5662.

### Statistical analysis

Results are presented as absolute numbers and percentages of respondents. Only participants who completed all questions about demographic data were considered valid for statistical analysis. The characteristics of respondents were compared according to answers to questions using Chi-square test or Fisher’s exact test.

To evaluate the predictors of responses to questions regarding the cause of persistent symptoms in patients treated with LT4, multivariate regression models were constructed. The dependent variable was the responses (on a scale from 1 -strongly disagree- to 5 -strongly agree-) to the question: “In most patients treated with LT4 who achieve normal serum TSH, persistent symptoms are due to” (question B16). The independent variables were sex (1-male, 0-female), age (< 40, 40–60 and > 60 years), years in medical practice (< 10, 11–30 and > 30), number of hypothyroid patients treated/year (< 50, 50–100 and > 100) and the cumulative responses to: “Thyroid hormones may be indicated in biochemically euthyroid patients with” (question B1) (sum of all “yes” answers in each respondent). All covariates with a p-value $<$0.10 in univariate analysis were introduced into a multivariate model. A backward elimination strategy was used to estimate the respective best model. All p-values are two-sided and values <0.05 were considered statistically significant. All analyses were conducted using IBM-SPSS® statistics software version 25 (SPSS Inc., Chicago-IL, USA).

## Results

### Sample characteristics

81 participants completed the questionnaire, which corresponds to 18.2% of the 446 LATS members. The demographic data of the respondents are shown in Table 1. The majority (91.3%) were endocrinologists, and 7 were endocrine surgeons specialized in thyroid surgery. Besides being members of LATS, 76.5% were members of a National Endocrine Society, whereas only six (7.4%) were members of the American Thyroid Association (ATA) and four (4.9%) of the European Thyroid Association (ETA). All the participants were clinically active.

**Table 1:** Characteristics of the respondents (n = 81)

	n (%)
Gender	
Female	55 (67.9)
Male	26 (32.1)
Age in years	
31–40	26 (32.1)
41–50	21 (25.9)
51–60	22 (27.2)
61–70	11 (13.6)
>70	01 (1.2)
Country of origin	
Brazil	51 (63)
Argentina	14 (17.3)
Chile	04 (4.9)
Others	12 (14.8)
Years in medical practice	
0–10	11 (13.6)
11–20	25 (30.9)
21–30	24 (29.6)
31–40	15 (18.5)
40 or more	06 (7.4)
Type of practice*	
University center	41 (50.6)
Regional hospital	24 (29.6)
Private clinic	64 (79)
Specialized medicine	10 (12.3)
Basic research	04 (4.9)
Society membership*	
American Thyroid Association	06 (7.4)
European Thyroid Association	04 (76.5)
National Endocrine Society	62 (76.5)
Other	47 (58)

*The sum of percentages exceeds 100% because some respondents

were employed in more than one place or were members of more

than one society

Most of the respondents (88.9%) treated thyroid patients on a daily basis, while 9 (11.1%) on a weekly basis. Fifty-seven members (70.4%) treated more than 100 hypothyroid patients annually, 15 (18.5%) managed 51–100 per year, 7 (8.6%) cared for 10–50 annually and only 2 (2.5%) reported that they rarely treated hypothyroid patients.

### Treating patients with thyroid hormones

LT4 was the first treatment of choice for hypothyroidism for all respondents. Few participants would also prescribe LT4 + LT3 combination, or LT3, in their daily clinical practice (6 and 3 respondents, respectively). Most respondents (93.8%) stated that their patients were receiving the LT4 formulation recommended by their clinician.

**Fig. 1:**
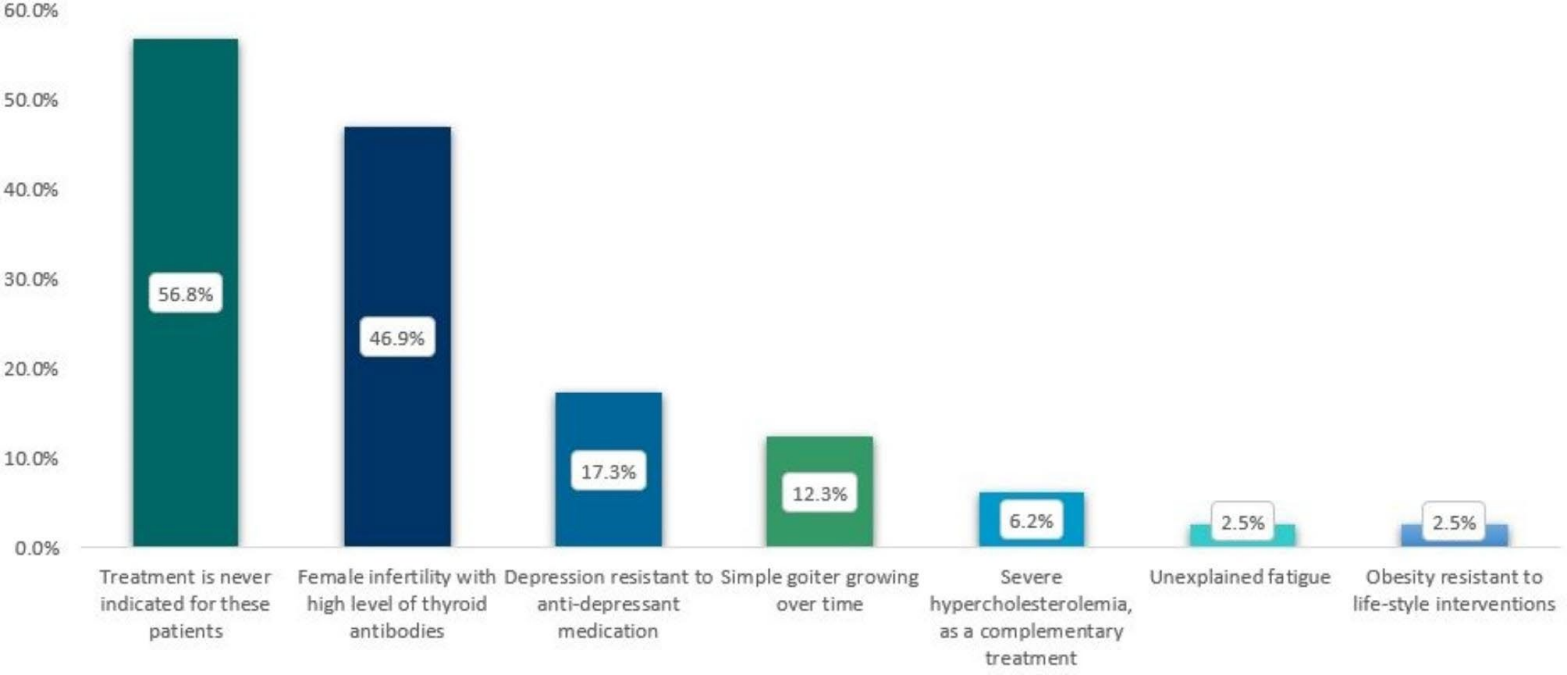
Reasons for prescribing thyroid hormones in euthyroid subjects. Multiple answers were possible

Forty-six of 81 respondents (56.8%) would prescribe thyroid hormones for patients with biochemically normal thyroid function in certain situations (Fig. 1). The two most frequently chosen indications were female infertility associated with elevated thyroid antibodies (38 of 81, 46.9%) and depression resistant to anti-depressant medication (14 of 81, 17.3%). Remaining indications are also shown in Fig. 1.

Female respondents were likely to suggest treatment with thyroid hormones for euthyroid female subjects with infertility associated with elevated thyroid antibodies (54.4% of women vs. 30.7% of men, p = 0.045). A similar, but non-significant trend (p = 0.06) was noted for respondents younger than 40 years old vs. those older than 40. Respondents who treated > 50 patients/year were more prone to consider thyroid hormone treatment for euthyroid infertile female subjects with positive thyroid antibodies, than those who treated < 50 patients/year (51.4% vs. 11.1%, p = 0.02). In contrast, 33.3% and 22.2% of respondents who treated < 50 patients/year would consider treatment in obesity resistant to lifestyle interventions or unexplained fatigue, respectively, vs. 2.8% and 0% treating > 50 patients/year (p = 0.03 and p = 0.008, respectively).

### Use of different LT4 formulations

Several questions explored scenarios in which alternative LT4 formulations (soft-gel capsules and liquid solution) might be preferred over LT4 tablets, although none of these formulations are currently available in Latin America. Table 2 shows respondents’ preferences in these situations. Assuming availability of all formulations, the majority of respondents would still recommend LT4 tablets. In case of food intolerance 20 of 81 (24.7%) would choose a liquid formulation and 39 of 81 (48.1%) a soft-gel or capsules/liquid solution. In patients who despite good biochemical control of hypothyroidism had persistent symptoms, 83.9% of respondents preferred tablets or expected no major changes with soft-gel capsules/liquid solutions (preferred by 16.1%; p < 0.001). No significant association was found between demographic characteristics of participants and formulation preferences.

**Table 2:** LT4 formulations preferred by respondents in different clinical scenarios

	Tablets, n (%)	Soft-gel capsules, n (%)	Liquid Solutions, n (%)	I expect no major changes with different formulations, n (%)
Interfering drugs may influence the stability of therapy. Which LT4 preparation is, in your experience, less likely to be subject to variable absorption?	33 (40.7)	20 (24.7)	19 (23.5)	9 (11.1)
Which of the following preparations of LT4 would you prescribe in case of a first diagnosis of hypothyroidism, when the patient self-reports intolerance to various foods, raising the possibility of celiac disease, malabsorption, lactose intolerance or intolerance to excipients?	37 (45.7)	16 (19.8)	20 (24.7)	8 (9.9)
Which of the following preparations of LT4 would you prescribe for a patient established on generic LT4 who has unexplained poor biochemical control of hypothyroidism?	32 (39.5)	18 (22.2)	17 (21)	14 (17.3)
Which of the following preparations of LT4 would you prescribe for a patient with poor biochemical control who is unable (due to busy lifestyle) to take LT4 fasting and separate from food/drink?	35 (43.2)	16 (19.8)	20 (24.7)	10 (12.3)
Which of the following preparations of LT4 would you prescribe for a patient established on generic T4 who has good biochemical control of hypothyroidism, but continue to have symptoms?	41 (50.6)	4 (4.9)	9 (11.1)	27 (33.3)

### Monitoring of thyroid hormone treatment

After initiating LT4 treatment for hypothyroidism, 41 of 81 (50.6%) physicians would re-check serum TSH level after 4 to 6 weeks, and the remaining (40 of 81; 49.4%) after 8 weeks (p = ns).

Most respondents would re-check serum TSH after 4–6 weeks (51.9%) or 8 weeks (38.3%) when switching to another formulation or changing to another manufacturer. If the dosage is unchanged, two members (2.5%) found no need for TSH monitoring and six members (7.4%) relied on clinical evaluation.

The responses were not significantly associated with any of the investigated demographic variables of the participant.

### Use of dietary supplements

Fifty-eight of 81 (71.6%) would never use supplementation with selenium or iodine in addition to thyroid hormone, while 23.5% (19 of 81) would consider this in case of coexisting autoimmune thyroiditis, and four (4.9%) in case of subclinical hypothyroidism. Use of supplementation was not significantly associated with any of the demographic variables.

### Combination therapy with LT4 and LT3

**Fig. 2:**
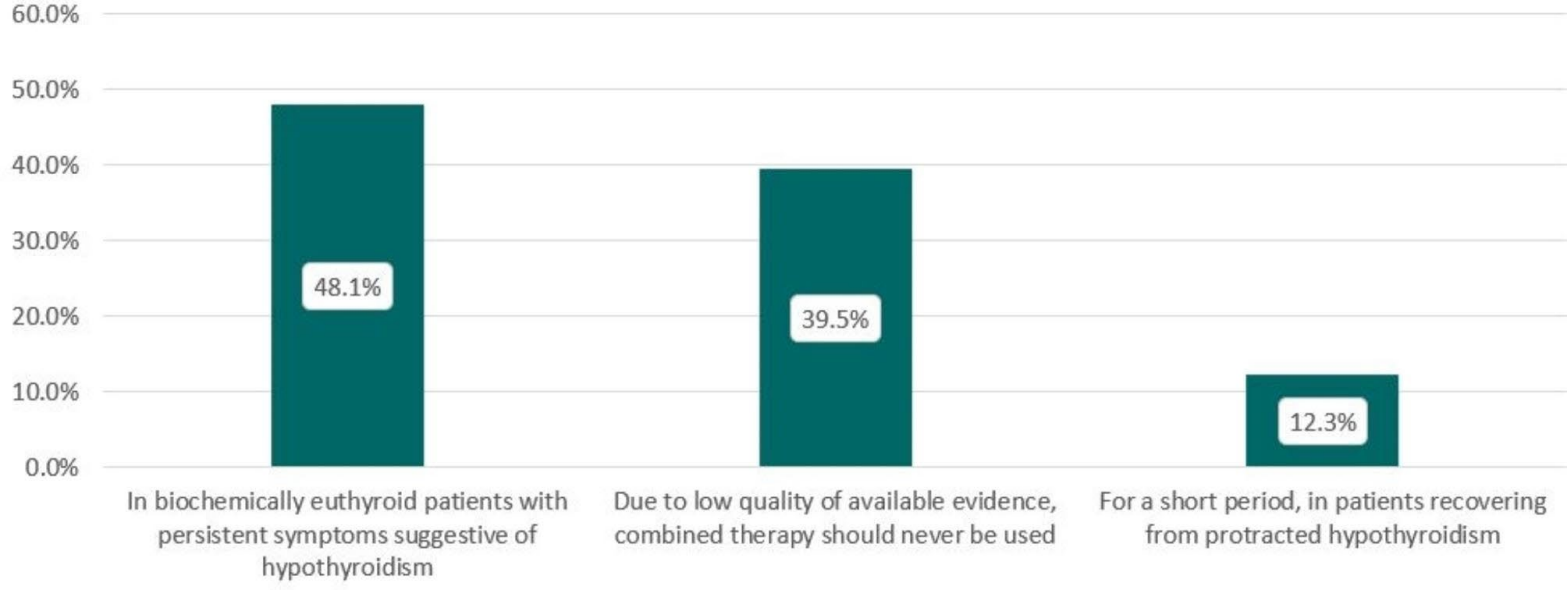
Use of levothyroxine (LT4) and liothyronine (LT3) combination therapy in different conditions

Combination therapy in biochemically euthyroid patients on LT4, and with persistent symptoms suggestive of hypothyroidism, was considered by 39 respondents (48.1%). Thirty-two (39.5%) would never use combination therapy, whereas 10 (12.3%) would recommend it for a short period in patients recovering from protracted hypothyroidism (Fig. 2).

### Persistent symptoms in LT-4 treated patients

Most of the respondents (61.7%) considered the percentage of symptomatic patients despite obtaining a normal TSH being less than 5%, or between 6 and 10% (29.6%). As for the trend over the past five years, 47 (58.0%) experienced no change, 18 (22.2%) saw more such cases, six (7.4%) observed fewer, and ten (12.3%) were not sure.

**Fig. 3:**
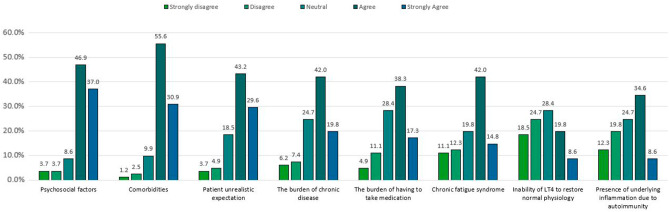
Possible explanations for persistent symptoms in hypothyroid patients who achieved normal TSH under LT4 treatment. The bars extend from strongly disagree (left), to strongly agree (right)

Participants were asked to comment on eight possible causes for persistent hypothyroid symptoms, with five options (strongly disagree, disagree, neutral, agree, strongly agree). The most likely explanations were comorbidities (86.4%), psychosocial factors (83.9%) and patients’ unrealistic expectations (72.8%) (Fig. 3).

### Respondents with diagnosed hypothyroidism

Sixteen of 81 participants (19.8%) declared having a diagnosis of hypothyroidism requiring thyroid hormone treatment. Three of them (18.8%) had experienced excessive tiredness/fatigue and 2 (12.5%) had tried LT4 and LT3 combination therapy, whereas no one had tried desiccated thyroid extract (DTE). Given a hypothetical scenario of respondents, 11 of 79 (13.9%) considered DTE or combination LT4 and LT3 as therapeutic options.

## Discussion

LATS was founded in 1974, with the goal of promoting “knowledge and research of the thyroid gland and its diseases” [36]. In 2013 LATS published a Clinical Practice Guidelines for the Management of Hypothyroidism, recommending levothyroxine as the drug of choice to treat hypothyroidism, while routine use of combined therapy was not endorsed [6].

This is the first survey of its kind in Latin America. Of note, unlike previous THESIS publications, which reflect mainly the opinions of physicians affiliated to national endocrine societies, our study represents the attitudes of LATS members that are highly specialized in thyroid pathology. In addition, they belong to different Latin American countries.

This could partially explain the differences in the response rates. However, key opinion leaders were among the respondents suggesting that this is representative of those teaching and producing the guidelines which are followed also by other specialties, such as GPs.

The predominance of female respondents (68%) accords with what is reported in most European publications. Most participants were specialists in endocrinology and treated a high volume of patients on a daily basis.

In accordance with current international and local guidelines [6, 37], and with the results of all the national THESIS studies published so far [13–32], LT4 was the treatment of choice for the treatment of hypothyroidism among LATS members.

### Monitoring of thyroid hormone treatment and combination therapy with LT4 + LT3

All respondents would re-check serum TSH level after 4–8 weeks, as recommended by guidelines, such as the one by LATS [6]. No one would rely only on clinical evaluation for monitoring therapy.

LT4 was the treatment of choice for hypothyroidism for all respondents, with none opting for LT3, LT4 + LT3 combination or DTE. Nevertheless, about 60% of participants would consider combination therapy with LT4 + LT3 for patients with normal serum TSH complaining of persistent symptoms suggestive of hypothyroidism, and less frequently, for protracted hypothyroidism. This accords with that seen in a number of other countries [21, 30, 32, 34], while this propensity of recommending combination therapy was least pronounced in Italy (41.4%) and Bulgaria (40%) [16, 17].

### Use of dietary supplements

Selenium supplementation decreases circulating thyroid autoantibodies in patients with chronic autoimmune thyroiditis [38]. However, whether this effect correlates with clinically relevant outcomes remains to be demonstrated [39]. In a survey of ETA members 80% responded that there is insufficient evidence for efficacy, but paradoxically 69% acknowledged having used selenium occasionally or routinely in euthyroid patients with antithyroid antibodies [40, 41].

70% of LATS respondents indicated that supplementation with selenium or iodine should never be used in addition to thyroid hormone replacement, which is close to the 60% figure of the Swedish THESIS survey [34], while the mean figure for all THESIS publications was 34.5%. About a quarter of LATS respondents considered supplementation in case of coexisting autoimmune thyroiditis compared to a mean of 19.2%,of other national THESIS publications (highest propensity found in Romania (56,8%) and lowest in Ireland (4%) [19, 35].

### Use of different LT4 formulations

For optimal efficacy, the LT4 tablet formulation requires avoiding concomitant ingestion of food, drink and certain medications, as well as excellent patient compliance. Common gastrointestinal disorders such as Helicobacter pylori infection, chronic atrophic gastritis, lactose intolerance, and some drugs have been reported to necessitate increased LT4 doses [15]. In recent years, alternative LT4 formulations, such as soft gel capsules and liquid solutions, have become available and have rapidly gained attention because of their pharmacokinetic properties [15, 42]. In selected categories of patients these new formulations have shown promise in improving treatment. However, they are more costly and unavailable in Latin America [43].

Assuming that all formulations were available, most LATS respondents preferred LT4 tablets. One in four would choose liquid solutions in case of food intolerance and almost half would consider soft-gel capsules/liquid solutions. These answers are in accordance with those obtained from most other European surveys, except the Italian THESIS investigation, where 75% recommended soft-gel capsules or liquid solution for patients established on LT4 with poor biochemical control of their hypothyroidism. The Italian data probably reflect long standing availability of these formulations [16] and intense marketing activity.

### Persistent symptoms in LT-4 treated patients

Most respondents found persistent symptoms in patients with hypothyroidism who achieve a normal TSH under medication to be an infrequent problem, affecting < 5% of their patients. Furthermore, the prevalence of patients with persistent symptoms was reported not to have changed over the last five years.

LATS members, in accord with other national THESIS investigations, indicated that the most common reasons for persistent symptoms in hypothyroid patients, despite normalization of TSH were comorbidities, psychosocial factors, and unrealistic patient´s expectations, rather than inability of LT4 to restore normal physiology. This impression is supported by a recent patient survey, which found a strong correlation between patient dissatisfaction with treatment and care and mistrust in healthcare professionals, while LT4 treatment (compared to LT3-containing regimens) was associated with a positive impact on daily living [44]. In addition, a high prevalence of somatization was noted among hypothyroid patients and a tendency to attribute all persistent symptoms to hypothyroidism or its treatment [45], implying that personality traits may influence the propensity of hypothyroid patients being dissatisfied with thyroid hormone treatment.

### Respondents with diagnosed hypothyroidism

Sixteen of 81 (19.8%) LATS respondents declared having a diagnosis of hypothyroidism requiring thyroid hormone treatment. Among them, only 3 reported persistence of symptoms such as fatigue, and only two were treated with LT4 + LT3 combination. This is remarkably at variance with half of them stating that combination therapy may be considered in various clinical scenarios, while just 16.9% would consider this treatment for themselves if they developed hypothyroidism.

### Treating hypothyroid and euthyroid patients with LT4

Although not recommended, more than half of the respondents would prescribe thyroid hormones for biochemically euthyroid patients in certain situations; the most frequent being female infertility associated with elevated thyroid antibodies (46.9%), potentially to prevent risk of miscarriage and preterm delivery [46, 47]. However, recent large-scale randomized studies found no impact of LT4 treatment on the rate of miscarriage, preterm delivery, or live births in euthyroid anti-TPO positive women [48, 49]. Interestingly, female LATS respondents were more supportive of treatment than their male counterparts. This trend was also observed in the Czech THESIS publication [21]. It could be hypothesized that female respondents were more likely to identify with this situation, also strengthened by this preference being more pronounced among respondents in the fertile age range (< 40 years).

Notably, 12% of LATS members considered the use of thyroid hormone treatment in patients with a simple goiter growing over time, compared to more than 40% in several other THESIS publications [17, 21, 23, 25, 30]. The high percentage of LATS respondents not supporting the use of thyroid hormones for simple goiter is in keeping with guideline recommendations based on evidence that most patients with goiter do not benefit from LT4 suppressive therapy [50] and the potential adverse effects [8].

In accordance with the other THESIS surveys, a minority of LATS respondents (especially those who treat small numbers of hypothyroid patients), considered thyroid hormone treatment in euthyroid individuals with unrelated illnesses (morbid obesity, severe depression, unexplained fatigue, and/or hypercholesterolemia). LATS’s Guideline recommends the screening of hypothyroidism in certain cases, in order to exclude the thyroid disease, although treatment is not recommended if patient is euthyroid [6]. For instance, there is recognized relation between hypothyroidism and dyslipidemia, hypothyroidism and detrimental effects on cardiovascular system [51–53]. The same relation has been not consistent regarding depression [54–56].

### Strengths and limitations

As a strength of our study the vast majority of participants were endocrinologists (91.3%), specialized in the management of thyroid disorders and key opinion leaders. Another strength is that this is the first THESIS study performed outside Europe, providing important information about hypothyroidism management in Latin America. The main limitation is the low response rate. It is likely that most of non-respondents did not treat patients, given that some LATS members are not clinically active. The questionnaire was distributed during the COVID-19 pandemic, which might have impacted response rates negatively [57]. Another limitation is that respondents are mainly derived from tertiary care or University based Endocrinologists, which may not reflect the practice of all physicians treating hypothyroidism.

## Conclusions

For Latin American thyroid specialists, LT4 tablets are the treatment of choice for hypothyroidism, even in the presence of conditions affecting its bioavailability. A significant proportion of LATS thyroid experts would consider LT4 treatment in specific groups of euthyroid patient groups, despite lack of evidence for efficacy, and in contrast with practice guidelines, but in accord with that noted in most national THESIS Surveys. Although recognizing low evidence for benefits of LT4 + LT3 in euthyroid symptomatic patients, almost half of specialists would consider this indication. LT4 treatment of infertile euthyroid women with thyroid antibodies and a wish for pregnancy is still considered by a substantial fraction of LA endocrinologists. In contrast to a number of European countries, Latin American respondents report a low and unchanged proportion of dissatisfaction among their patients over the last five years.

## Data Availability

The data supporting this study was available by the participants responding the questionnaire.

## Abbreviations

DTE: Desiccated thyroid extract
THESIS: Treatment of Hypothyroidism in Europe by Specialists:An International Survey
LT3: Liothyronine
LT4: Levothyroxine
TSH: Thyroid-stimulating hormone
